# Genetic Network Architecture and Environmental Cues Drive Spatial Organization of Phenotypic Division of Labor in Streptomyces coelicolor

**DOI:** 10.1128/mBio.00794-21

**Published:** 2021-05-18

**Authors:** Vineetha M. Zacharia, Yein Ra, Catherine Sue, Elizabeth Alcala, Jewel N. Reaso, Steven E. Ruzin, Matthew F. Traxler

**Affiliations:** a Department of Plant and Microbial Biology, University of California, Berkeley, California, USA; University of British Columbia

**Keywords:** division of labor, chemical gradients, siderophores, regulatory networks, multicellular bacterium, development, natural products, global regulatory networks

## Abstract

A number of bacteria are known to differentiate into cells with distinct phenotypic traits during processes such as biofilm formation or the development of reproductive structures. These cell types, by virtue of their specialized functions, embody a division of labor. However, how bacteria build spatial patterns of differentiated cells is not well understood. Here, we examine the factors that drive spatial patterns in divisions of labor in colonies of Streptomyces coelicolor, a multicellular bacterium capable of synthesizing an array of antibiotics and forming complex reproductive structures (e.g., aerial hyphae and spores). Using fluorescent reporters, we demonstrate that the pathways for antibiotic biosynthesis and aerial hypha formation are activated in distinct waves of gene expression that radiate outwards in S. coelicolor colonies. We also show that the spatiotemporal separation of these cell types depends on a key activator in the developmental pathway, AdpA. Importantly, when we manipulated local gradients by growing competing microbes nearby, or through physical disruption, expression in these pathways could be decoupled and/or disordered, respectively. Finally, the normal spatial organization of these cell types was partially restored with the addition of a siderophore, a public good made by these organisms, to the growth medium. Together, these results indicate that spatial divisions of labor in S. coelicolor colonies are determined by a combination of physiological gradients and regulatory network architecture, key factors that also drive patterns of cellular differentiation in multicellular eukaryotic organisms.

## INTRODUCTION

How groups of genetically identical cells give rise to organized patterns of differentiated cells with discrete functions is a central question of developmental biology. This self-organization requires coordination between cells, i.e., cells need to interpret where they are in relation to other cells and when to initiate their respective differentiation programs. This idea is captured by the concept of positional information proposed by Wolpert, in which cells acquire information from gradients of external signals or physiological cues such as oxygen or metabolic end products (1, 2), which are then translated into spatial developmental patterns (3–5).

Many bacteria engage in multicellular activities that enable them to carry out functions that are advantageous when undertaken by many individual cells. Examples of activities coordinated in such a way include the initiation of biofilm formation (6, 7), secretion of virulence factors (8, 9), luminescence (10, 11), and secretion of public goods, such as extracellular enzymes (12) and siderophores (13, 14). The majority of these cases rely on quorum sensing to ascertain the surrounding population density of clonemates and are limited in terms of defined spatial patterning. However, over the past decade, multiple studies have examined spatial multicellularity within colony biofilms formed on agar surfaces by organisms such as Bacillus subtilis and Escherichia coli (15–21). Recent work by Srinivasan and colleagues has elegantly demonstrated that phenotypic differentiation at the level of gene expression occurs in a spatiotemporal manner within B. subtilis colonies, with cells producing extracellular matrix located at the colony periphery, cells committed to forming endospores located near the surface of the colony interior, and motile cells found below the sporulating cells (17). Thus, phenotypic differentiation of cells within these colonies constitutes a division of labor in which subpopulations of cells are engaged in specific tasks. It is widely hypothesized that gradients of nutrient availability and correspondingly responsive genetic circuits drive this patterned differentiation (15–17, 21). While the genetic elements at play during this developmental process have been investigated, the role of the underlying gradients remains relatively unexplored experimentally.

**FIG 1:**
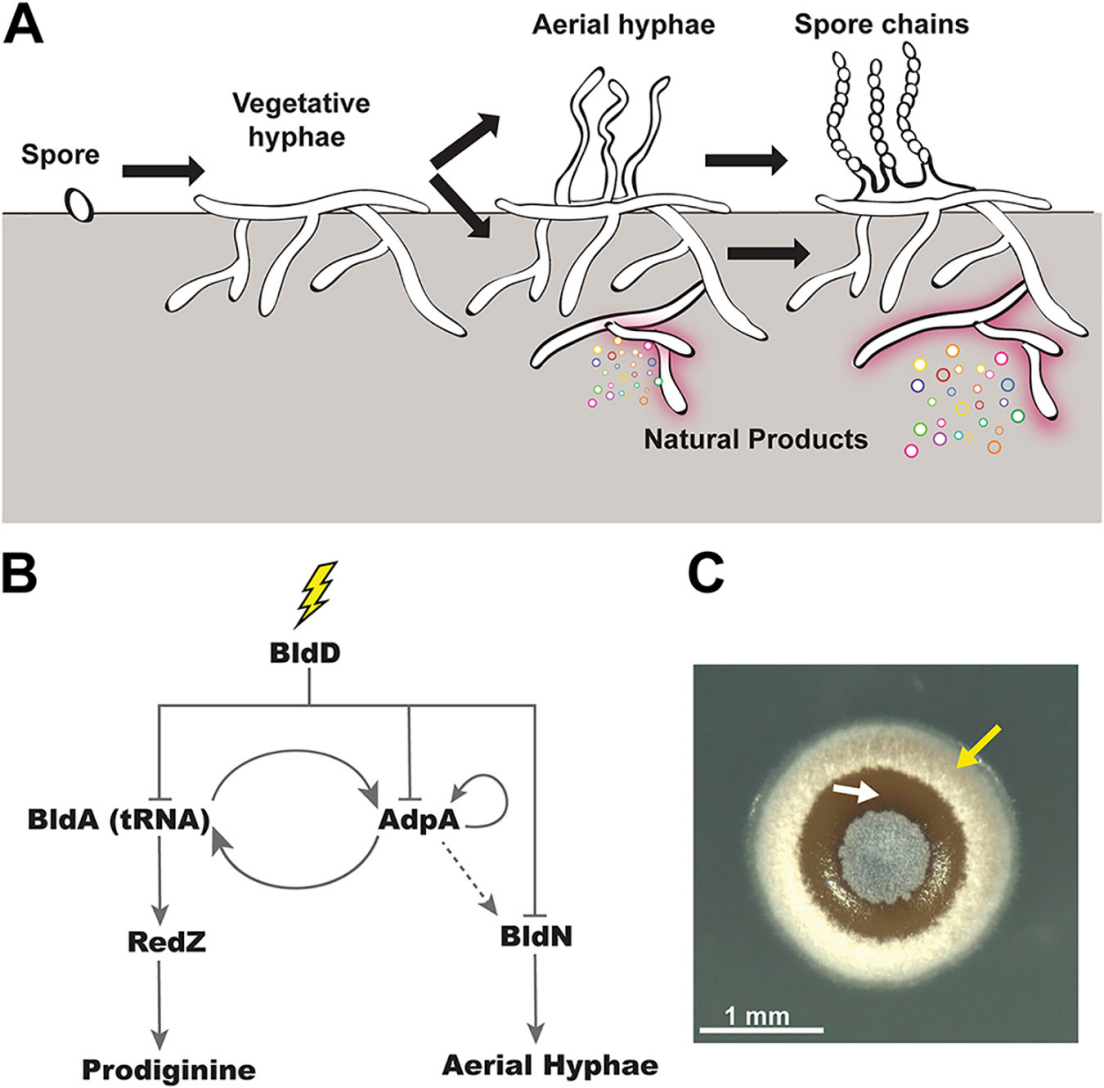
Regulatory network that determines cell fate in S. coelicolor. (A) The complex, multicellular life cycle of S. coelicolor begins once the spore germinates and grows as vegetative mycelium. Upon nutrient deprivation, S. coelicolor erects aerial hyphae and synthesizes natural products, such as undecylprodigiosin. Late in the life cycle, spore chains form and spores are dispersed. (B) Schematic of the simplified network architecture of S. coelicolor development and natural product biosynthesis. Upon external cues (lightning bolt), BldD derepresses master regulators BldA (tRNA) and AdpA, which regulate prodiginine biosynthesis and aerial hypha formation, respectively. AdpA is necessary for BldA transcription, and BldA tRNA is required for AdpA translation. Furthermore, AdpA positively autoregulates itself. The dashed gray arrow indicates indirect influence. (C) A top view of the S. coelicolor colony exhibiting white aerial hyphae (indicated by a yellow arrow) and red-pigmented undecylprodigiosin (white arrow). Scale bar, 1 mm.

Streptomycetes, including the model species Streptomyces coelicolor, have a life cycle that includes obligate multicellularity, making them attractive models for studying bacterial developmental processes (22–24). Upon germination, streptomycetes grow as a dense colony of interconnected filamentous cells known as a vegetative mycelium (Fig. 1A). In response to cues, like nutrient depletion, or signals that are not well understood, a subset of cells in the vegetative mycelium commit to a differentiation process that begins with the raising of hydrophobic aerial hyphae, which grow out of the colony surface into the air (25, 26). The distal end of each aerial hypha then undergoes a concerted round of genome duplication followed by septation, which yields a long chain of unigenomic spores (27, 28). Thus, these bacteria develop reproductive structures that are distinct from vegetative cells, and this differentiation has been suggested to be roughly analogous to the soma/germ division of multicellular eukaryotes (29). It has long been noted that, depending on the growth medium, aerial hyphae often seem to develop in organized patterns on the surfaces of *Streptomyces* colonies (30, 31), suggesting coordination of this developmental program at the colony level (Fig. 1C).

Streptomycetes are also important organisms from a medical standpoint, as they make an abundance of specialized metabolites, including antibiotics, that are used to treat human infections of many types (32, 33). An early observation in *Streptomyces* biology was that the induction of development (here referring to the program that produces aerial hyphae/spores), and the induction of specialized metabolism, including antibiotic production (34), often seem to occur around the same time, although in different places within the colony. This can easily be seen in the colony shown in Fig. 1C, in which the zones with fuzzy aerial hyphae and the areas of substrate mycelium producing pigmented antibiotics are distinct. Thus, the patterning of cellular functions in colonies of S. coelicolor is akin to the division of labor observed in B. subtilis biofilms, in which specialized cell types carry out specific functions or tasks that are beneficial for the colony as a whole (15, 29, 35). Recently, it was shown that S. coelicolor is also capable of a genetic division of labor in which a small percentage of cells irreversibly mutate, resulting in large-scale genomic deletions that trigger antibiotic production (36). However, how *Streptomyces* spatially organize phenotypic divisions of labor, wherein cell types are defined by their gene expression, at the colony level remains poorly understood.

In this report, we investigate how S. coelicolor initiates patterns of cellular differentiation at the colony scale. We used fluorescent promoter fusions and time-lapse microscopy to monitor two key cell fates, including antibiotic production and the initiation of development, which have historically been thought to occur concomitantly. Surprisingly, we found that while expression of both of these pathways began with waves that radiated outward from the center of colonies, they were spatiotemporally separated, with expression of antibiotic biosynthesis preceding the decision to develop aerial hyphae. When we mutated a gene encoding a key activator in the pathway for development (*adpA*), we found that this spatiotemporal separation was severely disrupted, with expression of the developmental pathway largely overlapping areas with expression of the antibiotic biosynthesis pathway. We also observed that expression of these pathways could be decoupled by chemical gradients from neighboring microbial colonies of different species. Finally, we found that disrupting gradients that form around S. coelicolor colonies led to disordered expression of these two pathways, and normal spatiotemporal expression could be largely restored by supplementing the growth medium with a siderophore. Collectively, these findings support a model in which physiological gradients, regulatory architecture, and chemical public goods (e.g., siderophores) underlie spatiotemporal division of labor within colonies of S. coelicolor.

## RESULTS

### The regulatory network that determines cell fate in S. coelicolor.

Many of the regulatory cascades that control development of aerial hyphae and antibiotic production, and connections between these cascades, have been mapped in the organisms S. coelicolor, *S. griseus*, and *S. venezuelae* (22, 37). BldD, AdpA, and tRNA^BldA^ are some of the key regulators that comprise the highest levels of the genetic network that coordinates development (Fig. 1B). BldD is the master repressor that inhibits expression of an array of genes involved in development and natural product biosynthesis (22). When an uncharacterized signal results in a drop in cyclic di-GMP (c-di-GMP), BldD dimers separate, leading to derepression of the *bldA* and *adpA* promoters (38, 39), whose gene products comprise the next level of regulation in this network. tRNA^BldA^ is required for translation of a subset of proteins involved in development/antibiotic synthesis, including AdpA. In turn, AdpA positively regulates *bldA* transcription (40). tRNA^BldA^ is also required for the translation of RedZ, a master regulator for the undecylprodigiosin biosynthesis pathway (41); thus, tRNA^BldA^ serves as an important genetic connection between the pathways for development and antibiotic production. AdpA is positively autoregulated in S. coelicolor (42) and also positively influences expression of *bldN*, which encodes a sigma factor essential for transcribing genes involved in the formation of aerial hyphae (43). The action of the regulators within this network likely shapes the fate of cells within S. coelicolor colonies with regard to the key functions of development and antibiotic production; however, transcriptional activity within the different sections of this network has not been mapped in a spatiotemporal way at the whole colony level.

### Division of labor during development in S. coelicolor is spatiotemporally separated.

**FIG 2:**
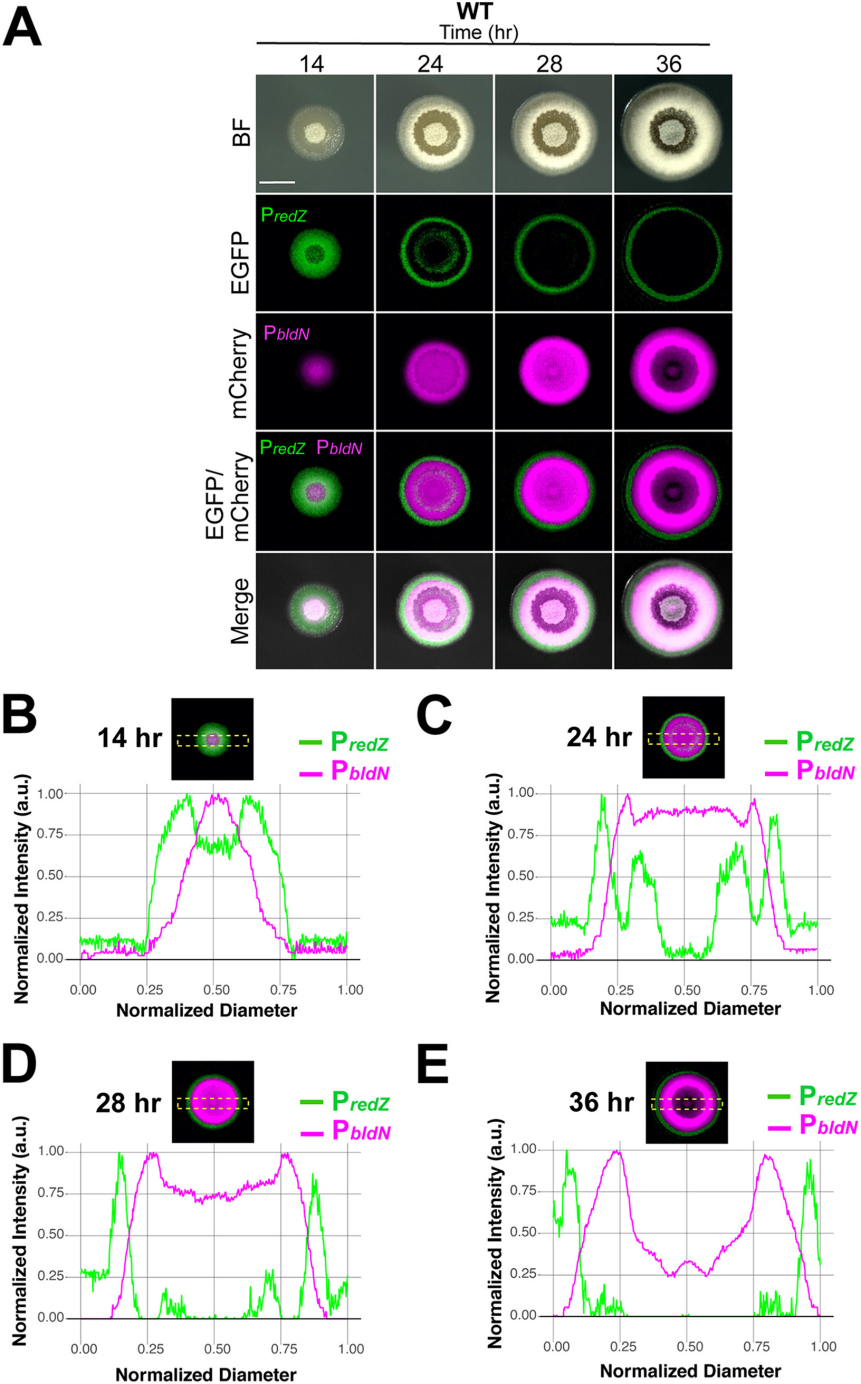
Division of labor during development in S. coelicolor is spatiotemporally separated. (A) Representative time-lapse micrographs of wild-type S. coelicolor containing dual fluorescent promoter reporters. P*redZ* expression (prodiginine biosynthesis) is localized over the entire colony at 14 h and radiates outward as the colony grows, followed by P*bldN* expression, associated with aerial hypha formation. (B to E) Normalized fluorescence intensity plot profiles of P*redZ* and P*bldN* expression at 14, 24, 28, and 36 h across the normalized colony diameter, corresponding to the yellow region of interest (ROI) box displayed in the merged EGFP/mCherry panels. Scale bar, 1 mm.

Based on the network architecture described above and a range of historical observations, we hypothesized that the activities of antibiotic biosynthesis and development would be partitioned into subpopulations of cells within S. coelicolor colonies. Thus, we sought to visualize activity within the corresponding areas of the network in growing colonies over time. To do so, we engineered key promoters within this network upstream of the fluorescent proteins enhanced green fluorescent protein (EGFP) and mCherry in vectors that integrate in single copy into different attachment sites in the S. coelicolor genome (see Fig. S1 at https://doi.org/10.6084/m9.figshare.14129516). We used these fluorescent reporter constructs to concurrently monitor transcriptional activity of combinations of two different promoters at the whole-colony level (Fig. S1). To do so, individual colonies of strains containing dual fluorescent reporters were grown on rich R2YE medium until they were approximately 500 μm in diameter and then monitored for at least 36 h using time-lapse fluorescence microscopy. Fluorescence intensity plot profiles were acquired using ImageJ software. Briefly, the intensity was calculated as an average intensity value per pixel of a region of interest (ROI), which spanned the colony diameter at the last (36-h) time point (Fig. 2B to E).

**FIG 3:**
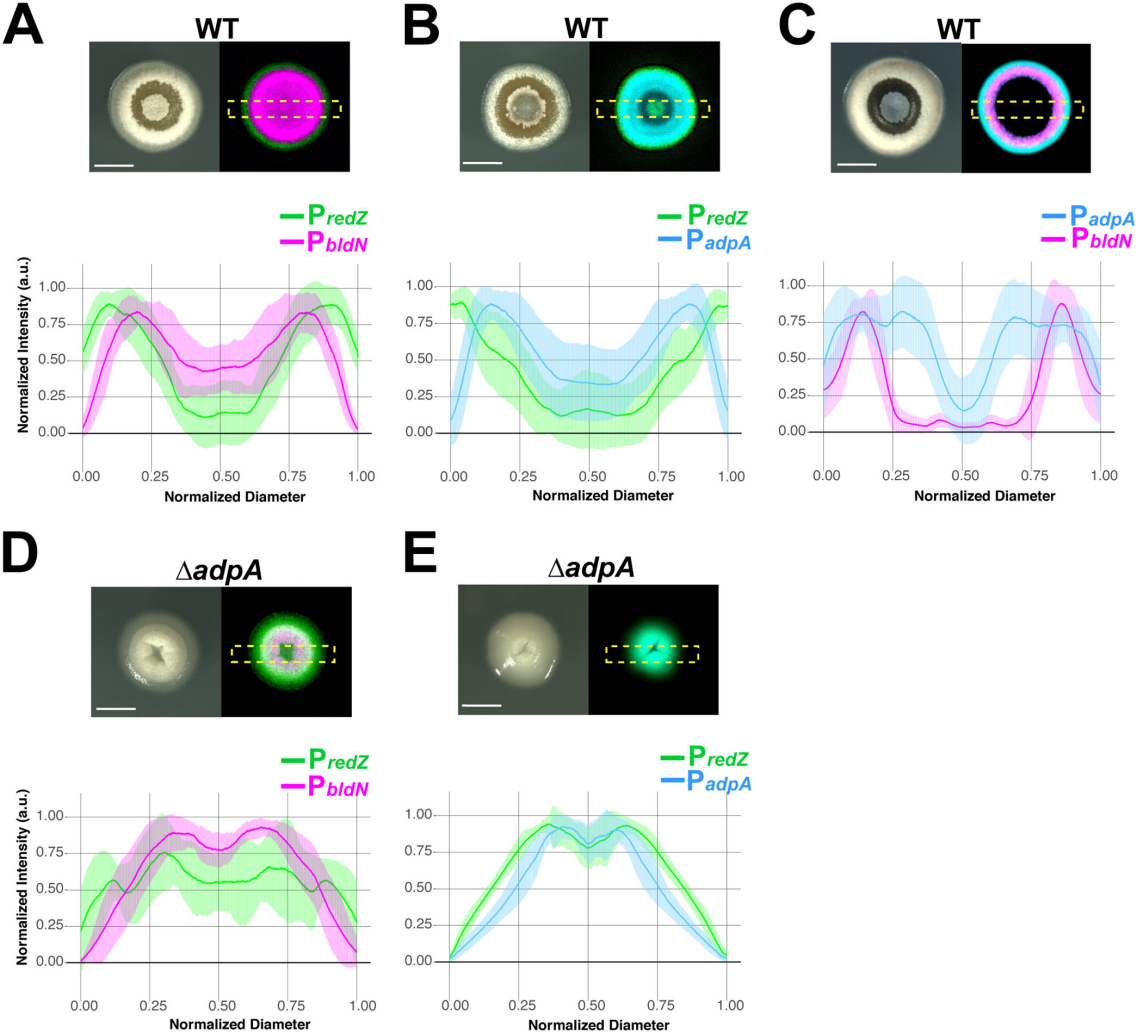
AdpA is required for the ordered pattern of gene expression in which *redZ* is followed by *adpA* and then *bldN*. (A) Representative micrographs (bright field and merged eGFP/mCherry) of WT S. coelicolor expressing P*_bldN_* and P*_redZ_.* Below the micrographs are individual plot profiles of eGFP and mCherry signals at 28 h (*n* = 16), normalized in amplitude of fluorescence intensity and colony diameter before averaging. (A to E) The shaded area corresponds to standard deviations. (A) The average plot profiles correspond to the same pattern seen in Fig. 2D, where *redZ* precedes *bldN* expression. (B) Expression of P*_redZ_* and P*_adpA_* in WT S. coelicolor, demonstrating that *redZ* precedes *adpA* expression. (C) WT S. coelicolor expressing P*_bldN_* and P*_adpA_*, where *adpA* radiates outward as the colony expands, followed by *bldN* expression, which is associated with aerial hypha formation (D) Δ*adpA* S. coelicolor mutant colonies expressing P*_redZ_* and P*_bldN._* In the bright-field (BF) channel, aerial hypha formation is reduced compared to that of the WT, and *adpA* expression is not spatiotemporally separated from *redZ* expression, as indicated by the overlapping eGFP/mCherry micrographs and plot profiles. (E) P*_redZ_* and P*_adpA_* expression in the Δ*adpA* strain overlap and are not spatiotemporally distinct, as demonstrated by the representative micrographs and plot profiles. Scale bar, 1 mm.

Our promoters of interest, in pairwise combinations, included *redZ* for initiation of prodiginine antibiotic biosynthesis and *adpA* or *bldN* to monitor the initiation of development. At the first time point (14 h), the S. coelicolor colonies contained a small central patch of white aerial hyphae surrounded by a “bald” ring of vegetative mycelium (Fig. 2A). We hypothesize that the central patch of aerial hyphae resulted from a round of development that occurred prior to the start of our visual/fluorescence monitoring. By 24 h into the time course, the colonies took on a complex morphology, which included a reddish hue in the colony interior, indicating the beginning of prodiginine antibiotic biosynthesis, a secondary ring of white aerial hyphae, and a ring of vegetative mycelium at the outermost edge of the colony (Fig. 2A; see Movie S1 at https://doi.org/10.6084/m9.figshare.14120456 for the full BF time-lapse). At the transcriptional level, P*_redZ_*-*efgp* expression (indicated by the fluorescence micrographs and plot profiles) radiated outward as the colony expanded (Fig. 2A to E and 3A and Movie S2 at https://doi.org/10.6084/m9.figshare.14120477), eventually being localized to the outermost ring of vegetative mycelium. Considering that RedZ is at the top of the multistep prodiginine biosynthetic pathway (44), it is logical that its induction preceded the appearance of red pigment in the colony. At 14 h, we observed a centralized peak of P*_bldN_-mcherry* expression, which then spread outward as a wave of expression whose peak followed behind the advancing apex of P*_redZ_*-*efgp* expression (Fig. 2A to E and Movie S2). Furthermore, at early time points (Fig. 2B and C), the highest fluorescent peak intensities were localized to the second and third quarters of the ROI, which corresponds to the center of the colony. At later time points (Fig. 2D and E), the signal peaks were localized to the first and fourth quarters, corresponding to the colony edge.

### Promoter activities of *redZ*, *adpA*, and *bldN* advance in discrete waves in a growing colony.

Since AdpA is situated near the top of the network that activates developmental pathways in S. coelicolor, we wanted to observe its expression pattern relative to *redZ* and *bldN*, which are further down in their respective regulatory cascades. Accordingly, we constructed fluorescent reporter strains that enabled visualization of P*_adpA_-mcherry* activity with either P*_bldN_-egfp* or P*_redZ_-egfp* activity. To demonstrate the robustness of the gene expression patterns, for each dual fluorescent reporter strain, we imaged *n* = 16 colonies at 28 h, since fluorophore signal intensity and peak separation were optimal at this time point. We note that the slight differences in colony appearance among the three fluorescent S. coelicolor strains in Fig. 2 and 3 can be attributed to minor variations in colony progression throughout the time course. Qualitatively, however, the growth/development patterns of all the strains were consistent in that colonies began with a small central patch of aerial hyphae and followed a similar developmental/antibiotic biosynthesis trajectory as described in the preceding section (Fig. 2A and 3A to C, BF panels). In the strain expressing P*_adpA_-mcherry* and P*_redZ_-egfp*, the P*_redZ_-egfp* signal peak localized to the colony edge and was spatially distinct from the peak of the P*_adpA_-mcherry* signal that followed behind it (Fig. 3B and Fig. S2 at https://doi.org/10.6084/m9.figshare.14129528 and Movies S3 and S4 at https://doi.org/10.6084/m9.figshare.14120471 and https://doi.org/10.6084/m9.figshare.14120474). In the P*_adpA_-mcherry-* and P*_bldN_-egfp*-expressing strain, the leading edge of the P*_adpA-_mcherry* signal radiated outward, with the peak of P*_bldN_-egfp* signal following closely behind (Fig. 3C and Fig. S3 at https://doi.org/10.6084/m9.figshare.14129537 and Movies S5 and S6 at https://doi.org/10.6084/m9.figshare.14120465 and https://doi.org/10.6084/m9.figshare.14120486). This pattern was expected, since *bldN* transcription is dependent (directly or indirectly) on AdpA (45). Taken together, the results presented in Fig. 2 and 3 indicate that expression of dedicated regulators for the pathways of antibiotic biosynthesis and development advance in waves whose peaks are spatiotemporally separated. Specifically, this ordered pattern of waves of gene expression begins with activation of *redZ*, followed by *adpA*, followed by *bldN* (Fig. 3A to C). At a broader level, this pattern of expression demonstrates that distinct subpopulations of cells within S. coelicolor colonies concurrently engage in different tasks, a hallmark of division of labor.

### AdpA is required for the spatiotemporal separation of developmental and antibiotic biosynthetic gene expression.

Given the key position of AdpA in the regulatory network governing development and that *adpA* expression is autoregulated, we hypothesized that a loss-of-function mutation in *adpA* would impact the timing of expression within the pathway for aerial hypha development. Using CRISPR-based genome editing, we generated an *adpA* loss-of-function mutant and found that the resulting mutant colonies failed to robustly produce aerial hyphae (Fig. 3D and E, BF panels; Fig. S4 at https://doi.org/10.6084/m9.figshare.14130614 and S5 at https://doi.org/10.6084/m9.figshare.14130620, Movies S7 and S9 at https://doi.org/10.6084/m9.figshare.14120468 and https://doi.org/10.6084/m9.figshare.14120462), which corroborated the bald phenotype of AdpA-deficient S. coelicolor strains seen in previous studies (42, 46). In the *adpA* mutant, the P*_redZ_-egfp* signal was not localized to the outermost regions of the colony over time but instead showed persistent strong expression near the colony center. This is seen in the plot profile peak intensities localized in the second and third quarters of the ROI (Fig. 3D and E; Fig. S4 and S5, Movies S8 and S10 at https://doi.org/10.6084/m9.figshare.14120480 and https://doi.org/10.6084/m9.figshare.14120483). When we visualized the expression of P*_bldN_-mcherry* in the Δ*adpA* strain (Fig. 3D; Fig. S4, Movie S8), we found that its peak expression was also localized primarily in the colony center (second and third quarters of the ROI). When we examined P*_adpA_-mcherry* expression in the Δ*adpA* strain, we found that it also showed peak expression localizing to the second and third quarters, a pattern that roughly paralleled the expression of P*_redZ_-egfp* (Fig. 3E; Fig. S5 and Movie S10). The restricted distribution of P*_adpA_-mcherry* expression in the Δ*adpA* strain indicates that autoactivation of the *adpA* promoter is critical for the robust, expanding expression observed in the wild type. A key result from this set of data is that the Δ*adpA* strain failed to spatiotemporally separate expression of the genes involved in development and antibiotic production; specifically, expression of the *redZ*, *adpA*, and *bldN* promoters all overlapped in the center of the colonies. Furthermore, these results imply that AdpA plays a role, either directly or indirectly, in downregulating expression of *redZ* in the interior of S. coelicolor colonies. Taken together, these results indicate that AdpA not only is required for robust expression of the developmental pathway but also is responsible for directing spatiotemporal separation of the division of labor in S. coelicolor colonies.

### Interspecies interactions can alter spatiotemporal *bldN* expression in S. coelicolor.

**FIG 4:**
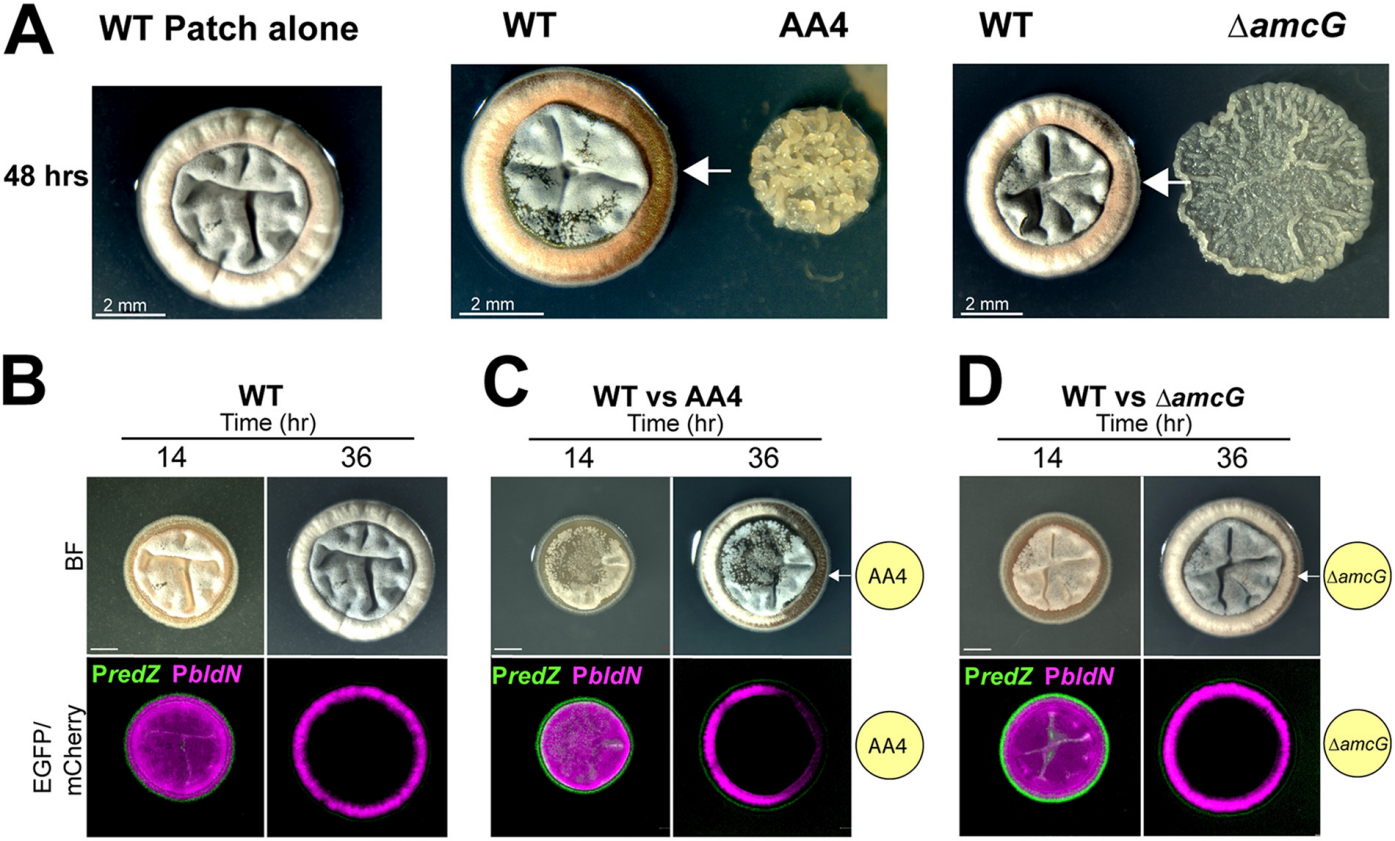
Interspecies interactions can alter spatiotemporal *bldN* expression in S. coelicolor. (A, left) Patch of wild-type S. coelicolor harboring the P*_redZ_*-*eGFP* and P*_bldN_*_-_*mCherry* plasmids grown alone and exhibits development and morphology similar to that of an individual colony. (Middle) When grown near wild-type AA4, S. coelicolor has reduced aerial hypha formation on the side that is closest to AA4 due to depleted iron levels (white arrow). (Right) S. coelicolor, when grown next to the Δ*amcG* mutant (amychelin-deficient AA4), has restored aerial hypha formation, indicating iron is important for aerial hypha formation. (B) Representative micrograph of S. coelicolor patch harboring P*_redZ_*-*egfp* and P*_bldN-_mcherry* plasmids when grown alone over 36 h. Similar to the wild type, the fluorescent micrographs of the S. coelicolor patch exhibit *redZ* preceding *bldN* expression. (C) S. coelicolor harboring P*_redZ_*-*egfp* and P*_bldN_*-*mcherry* plasmids, when grown next to AA4, has reduced aerial hypha formation (white arrow) and reduced P*_bldN_*_-_*mcherry* expression at the interaction interface, as shown in the representative fluorescent micrograph at 36 h. The yellow circle depicts the AA4-interacting strain for reference (not shown in the micrograph). (D) When grown next to the AA4 Δ*amcG* mutant, S. coelicolor P*_bldN_*-*mcherry* expression is restored, as is aerial hypha formation at the interaction interface (white arrow) at 36 h, as shown in the representative BF/fluorescence micrographs. The yellow circle depicts the Δ*amcG* AA4 mutant-interacting strain for reference (not shown in the micrograph). (B to D) Scale bar, 1 mm.

The soil plays host to an extraordinarily diverse microbial community. As a soil-dwelling bacterium, S. coelicolor likely encounters a wide range of microbial competitors, and prior work has demonstrated that these interactions can influence *Streptomyces* development and specialized metabolism (47–53). Our strains carrying dual fluorescent promoter fusions represented an opportunity to examine the transcriptional response at the spatial level during interspecies interactions. To do so, we recapitulated the conditions used in references 51 and 52, in which S. coelicolor development is curtailed when grown as a patch in proximity to another soil-dwelling actinomycete, *Amycolatopsis* sp. strain AA4 (Fig. 4A, captured 48 h into imaging). The ability of *Amycolatopsis* sp. strain AA4 to inhibit the formation of aerial hyphae by S. coelicolor stems from a depletion of iron, which *Amycolatopsis* causes by secreting the siderophore amychelin and by pirating S. coelicolor’s own siderophore, desferrioxamine E (DFO-E) (51, 52).

To remain consistent with previous interaction studies, we grew S. coelicolor as a patch, which was inoculated by adding ∼10^8^ spores in a 1-μl drop of liquid on an agar surface. S. coelicolor self-organization at the patch level was comparable to that of individual colonies in that there was a central region of aerial hyphae at 14 h that was followed by the formation of outer rings of aerial hyphae and vegetative mycelium at 36 h (Fig. 4B, BF panel, and Movie S11 at https://doi.org/10.6084/m9.figshare.14130563). At the level of promoter activity, the P*_bldN_-mcherry* and P*_redZ_-egfp* expression patterns were also spatiotemporally separated when an S. coelicolor patch was grown alone (Fig. 4B and Movie S12 at https://doi.org/10.6084/m9.figshare.14130566), and the order of expression was also maintained. As expected, when S. coelicolor was grown next to *Amycolatopsis* sp. strain AA4, the side adjacent to *Amycolatopsis* sp. strain AA4 exhibited reduced formation of aerial hyphae (Fig. 4A and C). Accordingly, the distribution of P*_bldN_-mcherry* signal was asymmetrical, with reduced expression at 36 h seen on the interacting side of the S. coelicolor patch (Fig. 4C and Movies S13 and S14 at https://doi.org/10.6084/m9.figshare.14130572 and https://doi.org/10.6084/m9.figshare.14130575). We next grew S. coelicolor next to a mutant strain of *Amycolatopsis* sp. strain AA4, which cannot make amychelin (Fig. 4D). In this interaction, by 36 h the symmetrical P*_bldN_-mcherry* expression pattern was largely restored, as was aerial hypha formation (Fig. 4D and Movies S15 and S16 at https://doi.org/10.6084/m9.figshare.14130578 and https://doi.org/10.6084/m9.figshare.14130581). These data provide spatial evidence that gradients of nutrient availability caused by neighboring microbes can disrupt normal patterns of ordered gene expression in S. coelicolor.

### Altering gradients, including gradients of siderophores, leads to disordered patterns of expression in S. coelicolor colonies.

**FIG 5:**
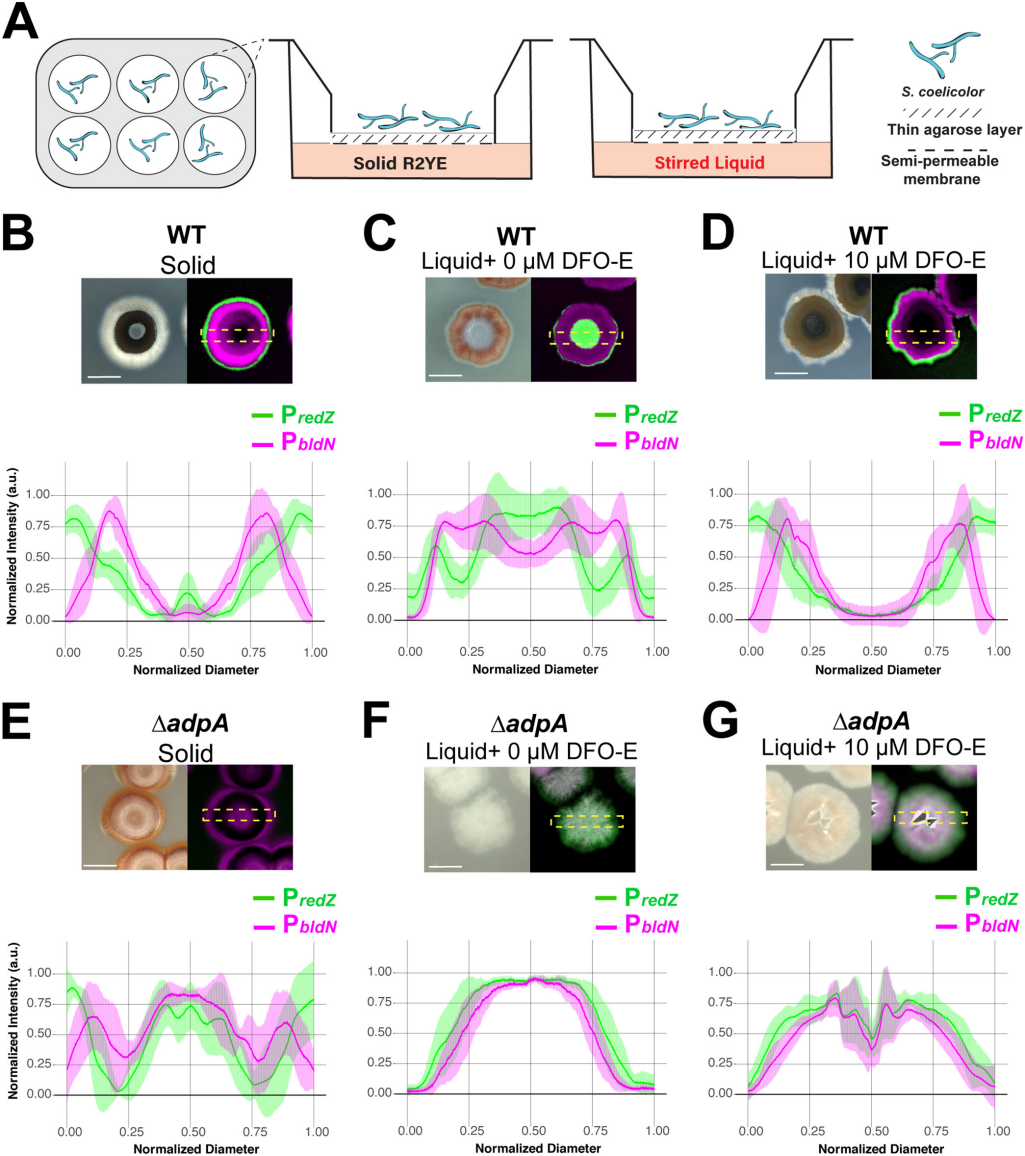
Altering gradients, including gradients of siderophores, leads to disrupted patterns of differentiation in S. coelicolor colonies. (A) Schematic illustrating a Transwell-based system such that gradients could be reduced and environmental conditions could be manipulated while allowing S. coelicolor growth. The semipermeable (0.4-μm-pore-size) membrane insert sits within tissue culture wells. A side view of the Transwell shows that solid or liquid medium is added to the bottom of the well, and directly above the medium is the semipermeable membrane with a thin (0.5%) agarose layer onto which S. coelicolor is spread. (B) Wild-type S. coelicolor harboring P*redZ*-*eGFP* and P*bldN*-*mCherry* plasmids grown on the permeable insert with solid R2YE in the well. S. coelicolor development and gene expression profiles recapitulate growth on solid agar plates, where *redZ* expression precedes that of *bldN* and *bldN* expression is associated with aerial hypha production. (C) S. coelicolor grown on the permeable insert with stirred liquid R2YE in the well to reduce fixed-concentration gradients of the S. coelicolor microenvironment. Shown is a representative BF micrograph showing altered S. coelicolor morphology and lack of aerial hypha formation when grown on liquid and altered *redZ* and *bldN* expression patterns. (D) S. coelicolor grown on the permeable insert with stirred liquid R2YE supplemented with 10 μM the siderophore desferrioxamine E (DFO-E). Aerial hypha formation is restored, as are spatiotemporally separated *redZ* and *bldN* expression patterns. (E) Representative micrograph showing Δ*adpA* S. coelicolor mutant grown under solid conditions with diminished aerial hypha formation and altered *bldN* and *redZ* expression patterns. (F, G) Under liquid conditions where gradients are reduced or supplemented with DFO-E, the Δ*adpA* S. coelicolor mutant fails to form aerial hyphae and has an altered colony morphology, and expression of *redZ* and *bldN* overlap, as shown in the representative micrograph and plot profile. Micrographs were taken 72 h postinoculation. Scale bar, 1 mm.

The concentric waves of gene expression we observed in S. coelicolor colonies share similarities to eukaryotic systems in which ordered patterns of cellular differentiation are directed by gradients. Previous work with *Bacillus* biofilms on agar surfaces has also led to the hypothesis that gradients of oxygen, nutrients, and signaling molecules influence the localization of specialized cell types (15, 17, 54, 55). However, empirical evidence supporting this idea is currently sparse. Thus, we sought to develop an experimental system that allowed us to disrupt gradients that form beneath S. coelicolor colonies while allowing the colonies themselves to grow intact on a solid surface. Previous work has demonstrated time-lapse imaging of *S. venezuelae* growth in a microfluidic device, since it can sporulate in liquid (56); however, growth on a solid support is critical for S. coelicolor, since it does not readily develop aerial hyphae or sporulate when grown in liquid media. To do so, we devised a Transwell (Corning, Inc.)-based system that allowed us to (i) grow the colonies on a semipermeable surface while providing access to nutrients from below and (ii) manipulate medium components to determine their possible effects on S. coelicolor division of labor. Specifically, we cultivated the colonies on a thin agarose layer inside a Transwell insert with a porous bottom, which could be placed either atop solid R2YE medium (where normal gradients would be expected to form) or into liquid R2YE medium that was agitated (where gradients would be disrupted) (Fig. 5A). Importantly, this setup also enabled S. coelicolor colony growth, development, and gene expression to be monitored from the top (Fig. 5A). For each Transwell condition, we imaged and averaged plot profiles of 8 colonies after 72 h of growth.

When colonies were grown with solid medium beneath the Transwell, the colony morphology was identical to when they were grown on solid agar plates, with the observed pattern of aerial hypha formation (e.g., a central patch and a secondary ring) and production of pigmented antibiotics (Fig. 5B, BF). Furthermore, P*_bldN_-mcherry* and P*_redZ_-egfp* each exhibited their normal order of expression and spatiotemporal separation (Fig. 5B). When stirred liquid medium was used in the bottom well, colony morphology was drastically altered such that there were no visible aerial hyphae and colonies appeared unusually flat (Fig. 5C, BF). Additionally, P*_bldN_-mcherry* and P*_redZ_-egfp* expression patterns were dramatically affected, with the strongest P*_redZ_-egfp* signal localized in the center of the colony and P*_bldN_-mcherry* expressed in a ring outside the region of P*_redZ_-egfp* expression (Fig. 5C, merged fluorescence channel). We interpret this altered pattern as an indication that local gradients formed within/beneath S. coelicolor colonies have a key role in driving typical patterns of cellular differentiation and downstream division of labor.

The interaction experiments in the preceding section demonstrated that P*_bldN_-mcherry* expression and formation of aerial hyphae were diminished due to iron depletion during a microbial interaction. We reasoned that when liquid medium was supplied under the Transwell insert, normal local concentrations of siderophores secreted by S. coelicolor might be diluted. Specifically, the siderophores might freely diffuse away from the colony, resulting in reduced iron uptake and, in turn, disrupting colony development, as seen in Fig. 5C. To test this hypothesis, we supplemented the liquid wells with 10 μM DFO-E. We found that added DFO-E partially restored key features of normal P*_bldN_-mcherry* and P*_redZ_-egfp* spatiotemporal expression and aerial hypha formation (Fig. 5D). Specifically, P*_redZ_-egfp* expression once again was located at the periphery of the colony, with a peak of P*_bldN_-mcherry* activity following the peak of P*_redZ_-egfp* expression. Additionally, the plot profile pattern was similar to that of the colonies grown under solid conditions.

Even though supplementation with DFO-E restored the key features noted above, subtle differences remained between these colonies and those grown on solid medium. For example, we noted two differences in the distribution of aerial hyphae on these colonies compared to the pattern observed on solid medium. Namely, the colonies lacked central patches of aerial hyphae, and the rings of aerial hyphae were less robust. We also supplemented the liquid wells with 100 μM FeCl_3_ and observed enhanced aerial hypha formation but unexpected distributions of P*_bldN_-mcherry* and P*_redZ_-egfp* expression patterns, possibly indicative of accelerated cycles of development and antibiotic biosynthesis (Fig. S6A at https://doi.org/10.6084/m9.figshare.14129546). We interpret these results as indicating that adequate access to DFO-E and, hence, iron is a prerequisite for normal patterning of division of labor within S. coelicolor colonies.

We next wanted to ask if the separation of gene expression for developmental and antibiotic biosynthesis pathways was AdpA dependent across the conditions used here. To do so, we grew the Δ*adpA* strain under these same conditions and examined the resulting patterns of P*_bldN_-mcherry* and P*_redZ_-egfp* expression. As expected, when grown in Transwell inserts placed on solid medium, the Δ*adpA* strain failed to produce aerial hyphae and had altered P*_bldN_-mcherry* and P*_redZ_-egfp* expression patterns (Fig. 5E). This included comparatively limited P*_bldN_-mcherry* expression and an atypical area of expression of both promoters in the center of the colonies. When liquid R2YE medium was included below the Transwell insert, Δ*adpA* colonies appeared flat, and expression of P*_bldN_-mcherry* and P*_redZ_-egfp* was broadly distributed across the colonies with little or no separation (Fig. 5F). Finally, when DFO-E was added to the liquid medium, there was neither the spatiotemporal separation of P*_bldN_-mcherry* and P*_redZ_-egfp* signals nor aerial hypha formation, indicating that DFO-E was not sufficient to restore these features in *adpA*-deficient S. coelicolor (Fig. 5G). Collectively, these results indicate that the spatiotemporal separation and patterning of antibiotic biosynthesis and aerial hypha formation are driven by the combined action of local concentrations of public goods (e.g., DFO-E), responsive genetic networks, and, to a lesser degree, other gradients that form in S. coelicolor colonies.

## DISCUSSION

**FIG 6:**
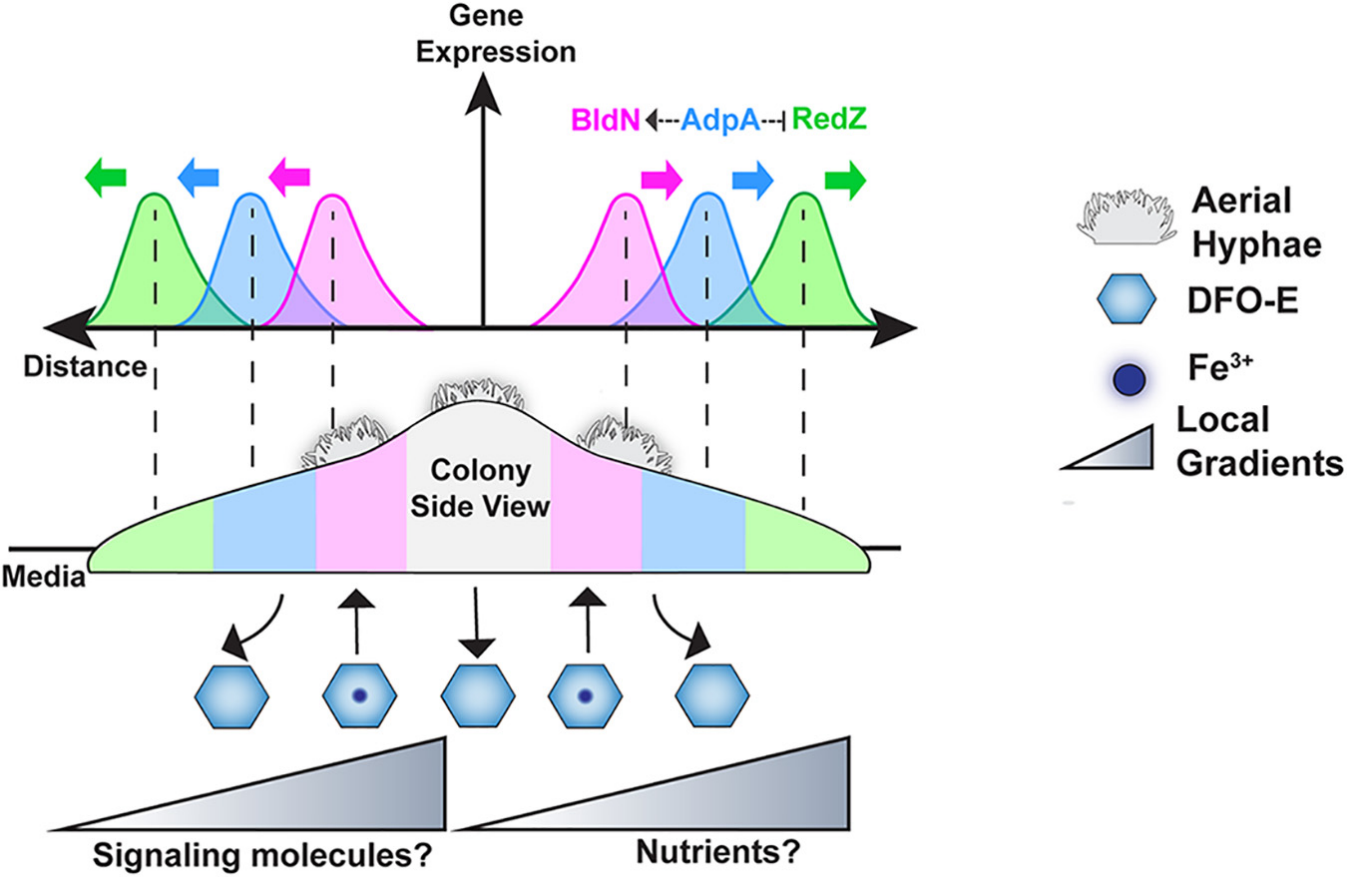
Regulatory networks and local gradients influence phenotypic division of labor in S. coelicolor. (A) Conceptual model illustrating S. coelicolor spatiotemporal organization of division of labor. In the cross-section view of a colony, P*_redZ_* expression localizes to the colony periphery, followed by P*_adpA_* expression, and, finally, P*_bldN_* expression radiating outward from the colony center. Individual peaks show spatiotemporal ordering between P*_bldN_* and P*_redZ_*, while the P*adpA* peak is between waves of P*_bldN_* and P*_redZ_* expression. AdpA directly or indirectly activates *bldN* and downregulates *redZ* expression. Waves of expression are influenced by gradients of signaling molecules or nutrients, specifically DFO-E.

Bacteria engage in a wide array of multicellular behaviors, ranging from formation of biofilms and production of virulence factors to coordinated synthesis of public goods (6–14). In some cases, such as in colony biofilms of Bacillus subtilis, subpopulations of cells are known to differentiate into phenotypic types that perform discrete functions, constituting a division of labor (15–21). While much is known about how these processes are regulated at the genetic level in B. subtilis, the extent to which other bacteria spatially organize such phenotypic divisions of labor, and how they may regulate any associated cellular differentiation, is poorly understood. Here, we report that within colonies of the multicellular bacterium S. coelicolor, different phenotypic subpopulations of cells are dedicated to the processes of antibiotic production or development of reproductive structures, as summarized in the conceptual model presented in Fig. 6. These two processes are activated in distinct waves of gene expression that radiate outward within S. coelicolor colonies. Specifically, we found that a wave of expression in the prodiginine antibiotic biosynthesis pathway, indicated by *redZ* promoter activity, preceded waves of expression in genes that initiate the developmental program, with activity from the *adpA* promoter occurring before activation of the *bldN* promoter (Fig. 2 and 3). Importantly, the spatial separation and ordering of this expression was dependent on both key regulatory network connections and environmental cues, including the local concentration of siderophores.

### Regulatory network architecture enables the spatiotemporal division of labor in S. coelicolor colonies.

How does regulatory architecture contribute to the spatial patterns of gene expression we observe in S. coelicolor colonies? The highest level of regulation in the S. coelicolor developmental network is the BldD system, whose repressive activity is controlled by cyclic di-GMP levels. However, the upstream stimulus that modulates the relevant diguanylate cyclases/phosphodiesterases that set these cyclic diGMP levels is unknown (22, 39, 57). We speculate that the stimulus monitored by the cyclic diGMP/BldD system is tied to the external gradients/environmental cues that arise within S. coelicolor colonies. The results presented here and elsewhere (52, 58) suggest that local iron/siderophore concentration should be considered an input of the BldD regulatory cascade. The other major pathway examined here, the prodiginine biosynthetic pathway, leads to production of antibiotics. The regulator RedZ is at the top of the regulatory cascade governing expression of the genes involved in prodiginine biosynthesis. Multiple regulators are known to influence *redZ* expression, including DasR (59), ScbR2 (60), and GlnR (61), among others (62–64). These regulators sense *N-*acetylglucosamine, antibiotics (including prodiginines and actinorhodin), and cellular nitrogen levels, respectively. Thus, these regulators, and gradients of their corresponding stimuli, are candidates for potentially driving *redZ* expression at the colony periphery.

Networks involved in spatial patterning often contain negative connections that serve to mutually exclude activity in potentially competing differentiation pathways (5, 65, 66). In S. coelicolor, AdpA is a key activator in the pathway for the formation of aerial hyphae and functions below BldD in this regulatory network. Previous reports have shown that *adpA* mutants exhibit abnormally high levels of prodiginine antibiotic production (42, 67). Thus, in addition to its developmental role, AdpA appears to play a negative role in regulating antibiotic biosynthesis. Here, we find that this dual regulation has spatial ramifications, with AdpA being required for the spatiotemporal separation of the waves of expression in the antibiotic and developmental pathways within S. coelicolor colonies. Specifically, rather than discrete waves of gene expression, the *adpA* mutant showed abnormal sustained expression of the prodiginine antibiotic biosynthesis pathway (P*_redZ_* expression), which overlapped expression in the developmental pathway (P*_bldN_* expression). This overlapping expression was aberrantly localized to the center of *adpA* mutant colonies (Fig. 3). These results indicate a role for AdpA in downregulating expression of the prodiginine antibiotic biosynthesis pathway to make way for the activation of developmental gene expression. The proposed negative effect of AdpA on *redZ* expression, and its positive effect on *bldN* expression, explains why *adpA* expression is spatially interposed between waves of P*_bldN_* and P*_redZ_* expression (Fig. 6). Moreover, this regulatory architecture provides a rationale for the well-developed boundary between cells expressing *redZ* and cells activating the developmental pathway observed in mature colonies after ∼28 h.

### Self-organization of S. coelicolor colonies requires normal local concentrations of siderophores.

Here, we present two lines of evidence that link the order of phenotypic differentiation in S. coelicolor colonies to local gradients of environmental cues. First, when S. coelicolor was grown next to another actinomycete known to deplete local iron concentrations, we found that the wave of expression in the developmental pathway in S. coelicolor colonies was not activated (Fig. 4). This finding indicates that expression of this pathway can be disrupted by external gradients of nutrient availability, specifically gradients of iron/siderophores. Moreover, it shows that the waves of gene expression we observe in these colonies can be differentially decoupled.

In another line of experiments, when we disrupted the gradients that normally form beneath growing S. coelicolor colonies (Fig. 5), colony morphology was altered in two key ways. First, the colonies did not form aerial hyphae. This is notable, since it indicates that when they were unable to form normal local gradients, the colonies incurred a fitness cost due to their inability to form spores. This developmental inhibition compromises both the ability to disperse and the ability to withstand adverse conditions. Second, when local gradients were disrupted, we discovered that expression patterns of both developmental and antibiotic biosynthesis pathways were dramatically disordered.

Supplementing the medium with a siderophore (desferrioxamine E) partially restored the typical ordered waves of expression within the developmental and antibiotic biosynthesis pathways (Fig. 5D). One interpretation of these findings is that in the experimental setup used here, the local concentration of siderophores was reduced through dilution when liquid medium was included below the Transwell insert. Based on this, we conclude that an adequate local concentration of siderophores/iron is a prerequisite for the typical ordering of phenotypic differentiation and division of labor within S. coelicolor colonies. This finding builds on previous work demonstrating a role for iron/siderophores in regulating developmental gene expression in these organisms (53, 58, 68, 69). We note that while major morphological features of colonies were restored by siderophore supplementation, subtle differences also remained in these colonies compared to ones that were grown on normal solid media. These differences suggest that gradients of additional signals/cues, or specific contours of siderophore gradients, are required to achieve the typical self-organization seen within these colonies.

### A common colony architecture across streptomycetes?

Why might colonies of S. coelicolor opt to spatiotemporally organize the expression of antibiotic biosynthesis and developmental genes as observed here? The nonmotile, filamentous lifestyle of streptomycetes ensures that they grow colonially, surrounded by their clonemates. This morphology likely provides advantages for nutrient foraging and acquisition in soil environments (29, 70), and, we speculate, increases the likelihood that local gradients will be formed and sensed at the colony level. However, this immobility may render filamentous actinomycete colonies susceptible to invasion by motile organisms. In such a scenario, it may be beneficial to dedicate cells on the periphery of a colony to production of antibiotics in an effort to protect public goods, like siderophores, for utilization by cells committed to development of aerial hyphae and spores.

Alternatively, it may be that the spatiotemporal separation of these divisions of labor is more important than the absolute configuration ultimately achieved. For example, it may be critical that areas of the colony first express antibiotic biosynthesis genes before they initiate the developmental process. This hypothesis aligns with recent work indicating that prodiginine production is part of a programmed cell death phase (71, 72). Beyond these possibilities, the recent findings described by Jones and colleagues of a novel explorer cell type, which respond to external cues and rapidly move across solid surfaces to explore new territory (73), hints that a variety of different cell types and further divisions of labor remain to be investigated in *Streptomyces*. The hypotheses above, combined with monitoring cell-specific expression markers as we have done in this work, form a framework for future investigations of *Streptomyces* spatial divisions of labor across new cell types, environmental conditions, and species. Finally, while we examined a phenotypic division of labor, genetic division of labor has also recently been described in S. coelicolor; thus, the potential combined impact of these two forms of specialization will be of notable interest for future study (36).

## MATERIALS AND METHODS

### Strains and media.

We grew S. coelicolor M145 on R2YE medium at 30°C. For conjugations, exconjugants were selected on mannitol soy (MS) agar plates containing nalidixic acid (25 μg/ml) and either apramycin (50 μg/ml) or hygromycin B (100 μg/ml) and streaked again on selective media to ensure growth of true exconjugants. To increase conjugation efficiency, MS agar plates containing apramycin were supplemented with 10 mM MgCl_2_ and 60 mM CaCl_2_. MS agar plates with hygromycin contained 10 mM MgCl_2_ to increase conjugation efficiency; however, CaCl_2_ was omitted, since a high salt concentration inactivates hygromycin activity. The conjugation donor strain used in this study was ET12567/pUZ8002. Genomic DNA from exconjugants was extracted and confirmed by PCR using primers found in Table S2 at https://doi.org/10.6084/m9.figshare.14206640 to verify the successful integration of plasmids and to ensure that native promoters of interest within the genome were not disrupted during plasmid integration. Plasmids and strains used in this study are found in Table S1 at https://doi.org/10.6084/m9.figshare.14206640.

### Plasmid and strain construction.

All plasmids containing EGFP were based on the pIJ8660 backbone vector (74) containing the phi-C31 *int* gene and *attP* integration site. Primers used in this study can be found in Table S2. To reduce extraneous mRNA transcripts of EGFP and enhance its expression, the RNA element, RiboJ, followed by a strong synthetic ribosome binding site, SR-41 (75), were introduced as a gene block with 5′ XbaI and 3′ NdeI ends into pIJ8660 at the multiple cloning site via restriction digestion and ligation. Promoters for *bldN* and *redZ* were amplified from M145 genomic DNA using primers engineered with 5′ EcoRV cut site and 3′ XbaI cut sites. The promoter fragments for *bldN*, *redZ*, and *adpA* were digested and introduced into the EcoRV- and XbaI-digested vector to generate pVZ027, pVZ056, and pVZ059, respectively. All mCherry-containing vectors were based on pAV-1 backbone (76), which contains the VWB *int* gene and *attP* integration site. The hygromycin B resistance cassette and the *bldN*, *redZ*, and *adpA* promoters were amplified using Gibson assembly-compatible primers and were introduced into the vector using the Gibson assembly mastermix (New England Biolabs) to create pVZ150, pVZ157, and pVZ216 plasmids, respectively. For a promoterless mCherry negative-control vector, a multiple cloning site was amplified using Gibson primers and introduced into the backbone to create pVZ177. All plasmids were transformed into the conjugation donor strain ET12567/pUZ8002.

### Constructing the *adpA* mutant.

To generate the *adpA* mutant strain, we adapted the protocol from Cobb and colleagues using the CRISPR/Cas-based gene editing optimized for *Streptomyces* species (77). Briefly, the S. coelicolor *adpA* mutant was engineered using the pCRISPomyces-2 plasmid (77). The online tool CRISPy-web (78) was used to select a suitable spacer sequence (Table S2) within the *adpA* gene. To reduce the potential of nontarget effects, the spacer sequence and its PAM sequence were searched through the S. coelicolor genome using BLAST to ensure a single optimal target site. The primer sequences for the spacer and editing templates are found in Table S2. The deletion introduces a premature stop codon 49 bp into the *adpA* gene, creating a nonsense mutation. Exconjugants were grown at room temperature, and replica plating was used to confirm that strains were apramycin sensitive. Promoter-fluorescent reporter plasmids of interest were conjugated into the *adpA* mutant background strain.

### Time-lapse microscopy.

S. coelicolor colonies were streaked out for isolation on freshly poured R2YE plates and incubated at 30°C for 48 h prior to imaging. Due to the presence of various colonies at different life cycle stages represented on a single plate, developmentally young colonies were selected (average diameter, 500 μm) for imaging. Time-lapse movies were recorded by taking images every 30 min for 36 h (after the 48-h incubation period) using the Zeiss Axio Zoom V16 microscope with a PlanNEOFLUAR Z 1.0× objective coupled with an AxioCam 506 color camera. The fluorescent light source used was the Illuminator HXP 200 C (metal halide), and the filter sets to capture EGFP and mCherry fluorescence images were FS 38HE (excitation BP, 470/40; emission BP, 525/50) and FS 43HE (excitation BP, 550/25; emission BP, 605/70), respectively. To maintain a 30°C temperature for optimal colony growth during the time-lapse, we developed a growth chamber. First, a temperature-controlled transparent heated lid (Tokai Hit) was placed on top of the petri dish with S. coelicolor colonies to be imaged. The petri dish was then placed on top of a USB-controlled heated mug warmer (Amazon), which was unplugged once the temperature equilibrated to 30°C. The mug warmer equilibrated the temperature and prevented condensation from forming on the transparent heated lid. Finally, a temperature sensor (Tokai Hit) was placed below the petri dish to monitor real-time temperature of the growth chamber and that it was maintained at 30°C throughout the time-lapse.

### Transwell setup.

To manipulate nutrient gradients, we developed a system in which we could grow S. coelicolor colonies on top of solid or liquid media using semipermeable membrane inserts (Corning 24-mm Transwell with 0.4-μm pore polycarbonate membrane). One milliliter of 0.5% agarose was pipetted onto the Transwell membrane to form a thin layer onto which S. coelicolor colonies (2 × 10^8^ spores/ml serially diluted) could be spread (approximately 20 colonies/well). Below the insert, 2 ml of solid or liquid R2YE was pipetted into the tissue culture well. In the liquid wells, one 3-mm-diameter glass bead (Fisher Scientific) was carefully inserted below the insert using forceps to allow for even mixing of liquid media in the shaking incubator. FeCl_3_ or DFO-E (nocardamine; Abcam) was added to the liquid wells at the indicated concentrations. Plates were placed in a 30°C shaking incubator at 125 rpm for 72 h before imaging.

### Interactions.

For interactions, 1 μl of strains from frozen spore suspensions were inoculated onto R2YE agar 5 mm apart from each other, and 1 μl of S. coelicolor spore stock (3 × 10^8^ spores) was inoculated and grown alone on R2YE solid agar as a control. Wild‐type *Amycolatopsis* sp. strain AA4 and Δ*amcG* strain colonies were grown by spotting 1 μl of spore stock (8 × 10^7^ spores) in 25% glycerol onto R2YE. Plates were incubated at 30°C for approximately 24 h before time-lapse imaging.

### Image processing.

To automate the processing of multiple, large time-lapse movies of fluorescent colonies to a negative-control colony (instead of only normalizing to the image background), we developed scripts (source code available at https://github.com/vzach1/Normalizing_Fluorescence_Time-lapse/blob/master/Zacharia_CB_Supplemental/Zacharize_Subtract%20FirstMean%20from%20SecondDataSet.txt). Mean intensity values for the negative-control colony (S. coelicolor colony integrated with promoterless EGFP and mCherry constructs) and fluorescent S. coelicolor colonies for each time point were calculated. The autofluorescence from the negative-control colony at each time point was subtracted from the signal of the fluorescent S. coelicolor colony to yield the final image. These images, in which background EGFP and mCherry signal were normalized to the negative control, were further processed. Briefly, we developed a script in ImageJ to automate the process of subtracting any remaining image background noise from the autofluorescence subtracted colony (source code available at https://github.com/vzach1/Normalizing_Fluorescence_Time-lapse/blob/master/Zacharia_CB_Supplemental/subtractbg_loop_vz_saveas_noselectnone.txt). To control for gene expression variation due to different S. coelicolor genomic integration sites and fluorescent reporters, each promoter of interest was constructed upstream of either the EGFP phi-C31 integrating plasmid or the mCherry VWB-integrating plasmid and expression patterns were assessed (data not shown). Table S3 at https://doi.org/10.6084/m9.figshare.14206640 provides descriptions of supplemental movies. Images were normalized and processed using iVision, ImageJ, and Zen Blue. Final brightness and contrast values were adjusted linearly, and final figures were made in Adobe Illustrator. Plot profile graphs were generated and visualized using the ggplots2 feature in Rstudio.
